# Supplementary material for: MYC reshapes CTCF-mediated chromatin architecture in prostate cancer
Source: Nat Commun. 2023 Mar 30;14:1787. doi: 10.1038/s41467-023-37544-3 (PMC10063626; doi:10.1038/s41467-023-37544-3)

SUPPLEMENTARY INFORMATION

MYC reshapes CTCF-mediated chromatin architecture in prostate cancer

Wei *et al*

Inventory of Supporting Information

Supplementary Figure 1-6

Supplementary Tables 1-5

Uncropped scans of blots and gels in Supplementary Figures

Supplementary Figure 1

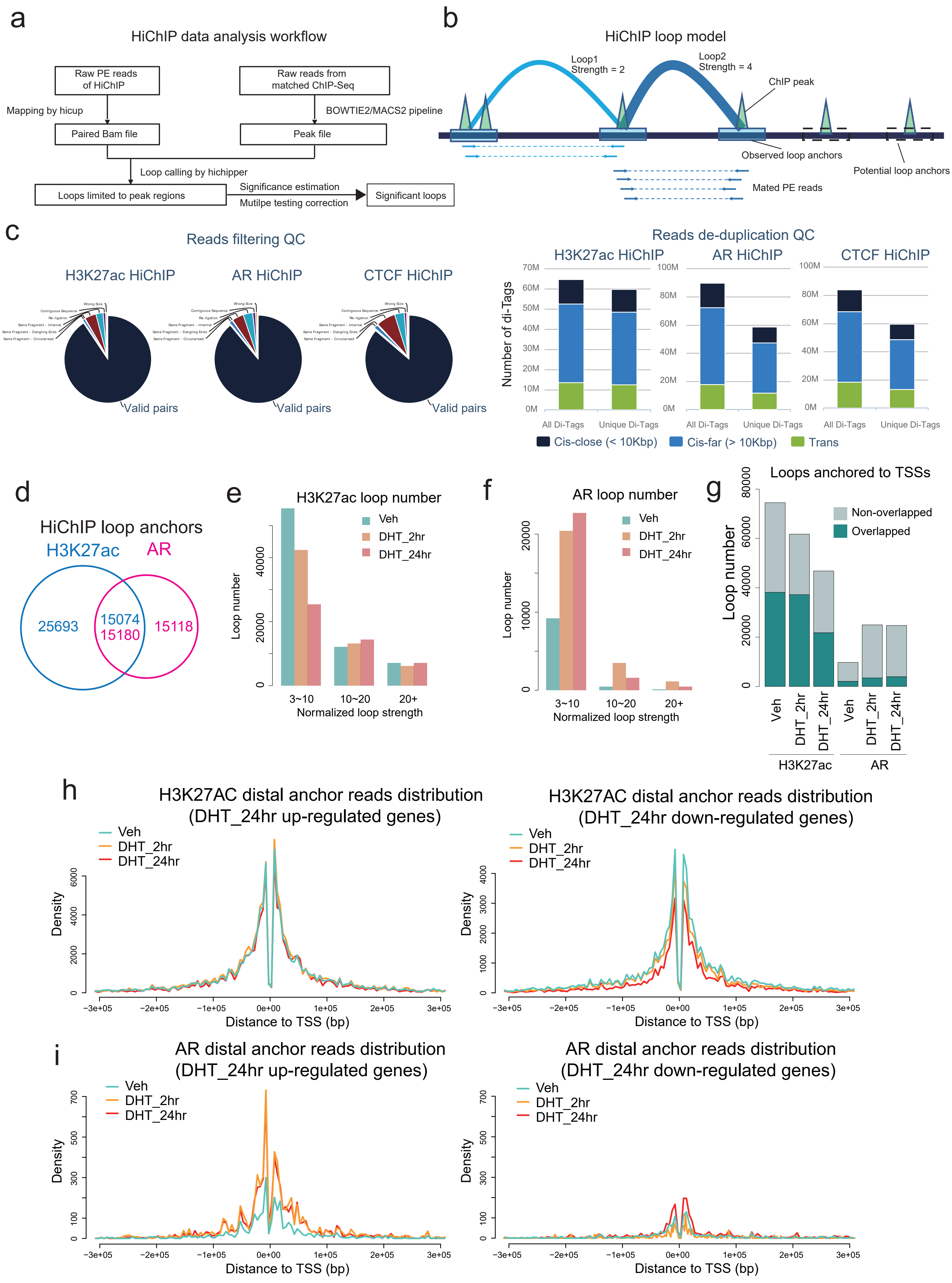

Supplementary Figure 1. 3D epigenomic profiles of PCa cells.

(a) Illustration of HiChIP data analysis workflow in this study.

(b) Illustration of the HiChIP loop model used to find the significant loops.

(c) HiCUP QC reports of di-tag filtering and de-duplication results. The shown samples are H3K27ac, AR and CTCF HiChIP libraries in VCaP cells under DHT condition.

(d) Venn diagram showing the overlapping between H3K27ac HiChIP loop anchors and AR HiChIP loop anchors.

(e) H3K27ac loop strength distribution showed DHT stimulation decreased the number of H3K27ac loops with low strength. From left to right, n = 55374, 42414, 25374, 12110, 13170, 14395, 7030, 6164, 7050, respectively.

(f) AR loop strength distribution showed DHT stimulation increased the number of AR loops with both low and high strength. From left to right, n = 9211, 20402, 22698, 449, 3467, 1583, 91, 1113, 456, respectively.

(g) Summary of the fractions of H3K27ac and AR-associated loops anchored or not anchored to the TSSs of expressed genes. Expressed genes were defined as genes of average FPKM > 0.1 in VCaP cell RNA-Seq data. From left to right, n = 74514, 61748, 46819, 9751, 24982, 24737, respectively.

(h) The H3K27ac loop strength was decreased in genes downregulated by 24 hr DHT treatment but not in genes upregulated by 24 hr DHT treatment. For TSSs of upregulated and downregulated genes, n = 1398 and 1585, respectively.

(i) The AR loop strength was increased in genes upregulated by 24 hr DHT treatment and was very weak in genes downregulated by 24 hr DHT treatment. For TSSs of upregulated and downregulated genes, n = 1398 and 1585, respectively.

Supplementary Figure 2

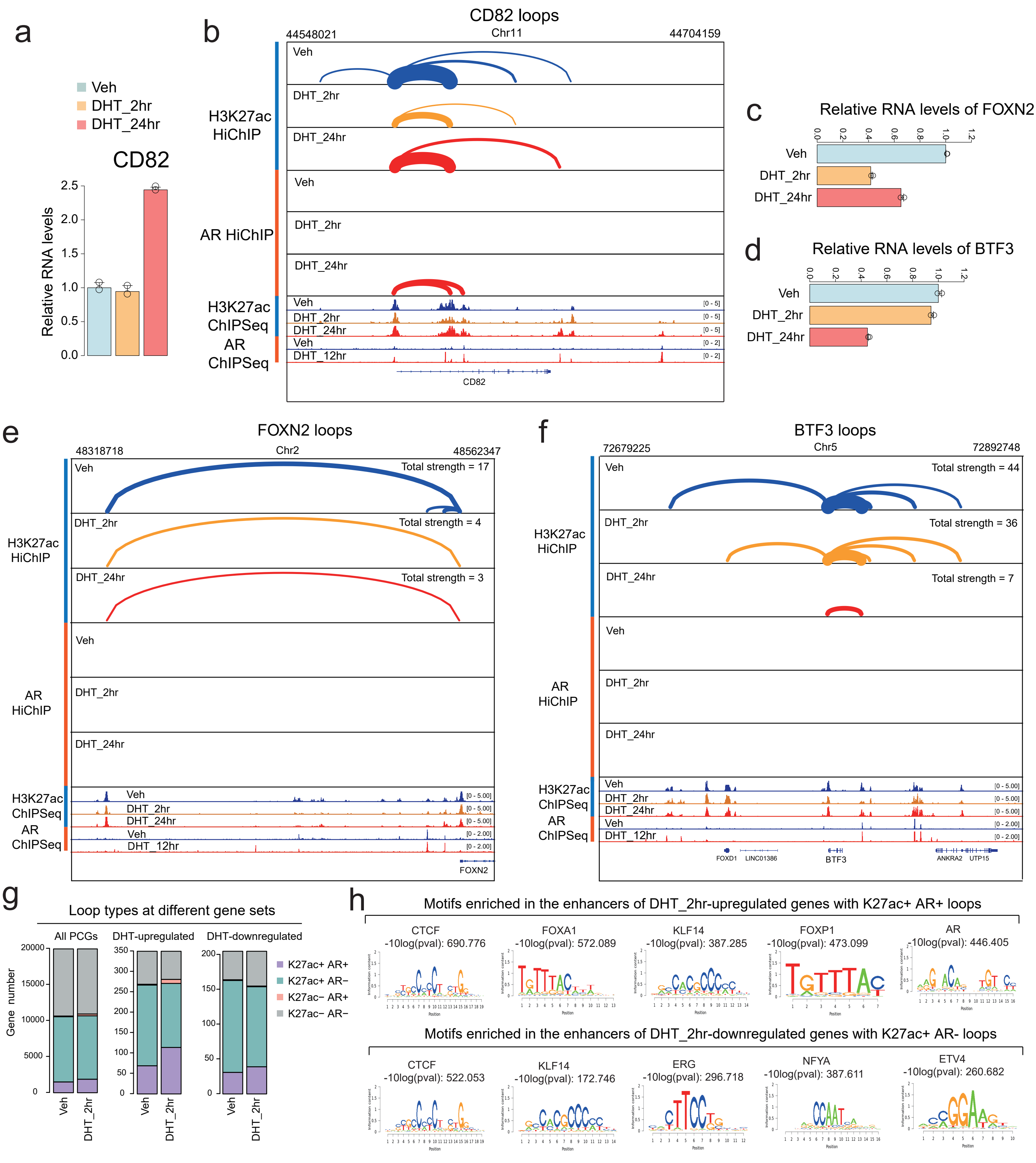

Supplementary Figure 2. AR and H3K27ac HiChIP analyses in DHT-treated VCaP cells.

(a) The expression of CD82 was upregulated only at the late time point (24 hr) after DHT stimulation. n = 2. Data represent means ± SD.

(b) For the CD82 gene, which was induced by DHT at 24hr, AR loops were boosted at 24 hr but not 2 hr after DHT stimulation.

(c) FOXN2 expression was repressed from the early time point after DHT stimulation. n = 2. Data represent means ± SD.

(d) BTF3 expression was repressed only at the late time point after DHT stimulation. n = 2. Data represent means ± SD.

(e) For FOXN2 gene, H3K27ac loops were diminished as early as 2hr after DHT stimulation. No AR loop was observed in the FOXN2 gene. For normalized H3K27ac loop read counts, n = 17, 4, 3, from top to bottom, respectively.

(f) For BTF3 gene, H3K27ac loops were diminished at 24hr but not 2hr after DHT stimulation. No AR loop was observed in the BTF3 gene. For normalized H3K27ac loop read counts, n = 44, 36, 7, from top to bottom, respectively.

(g) Summary of genes classified by the tethering of H3K27ac loops or AR loops at TSSs. For example, if there are both H3K27ac loop and AR HiChIP loop anchored at the TSS of a gene, this gene is classified as K27ac+ AR+ gene.

(h) Top motifs enriched in the open chromatin regions in the anchors of indicated loops. The distal anchors of indicated HiChIP loops (H3K27ac or/and AR loops) were intersected with TCGA PRAD ATAC-Seq peaks, and the overlapped ATAC-Seq peaks were used for motif enrichment analysis.

Source data are provided as a Source Data file.

Supplementary Figure 3

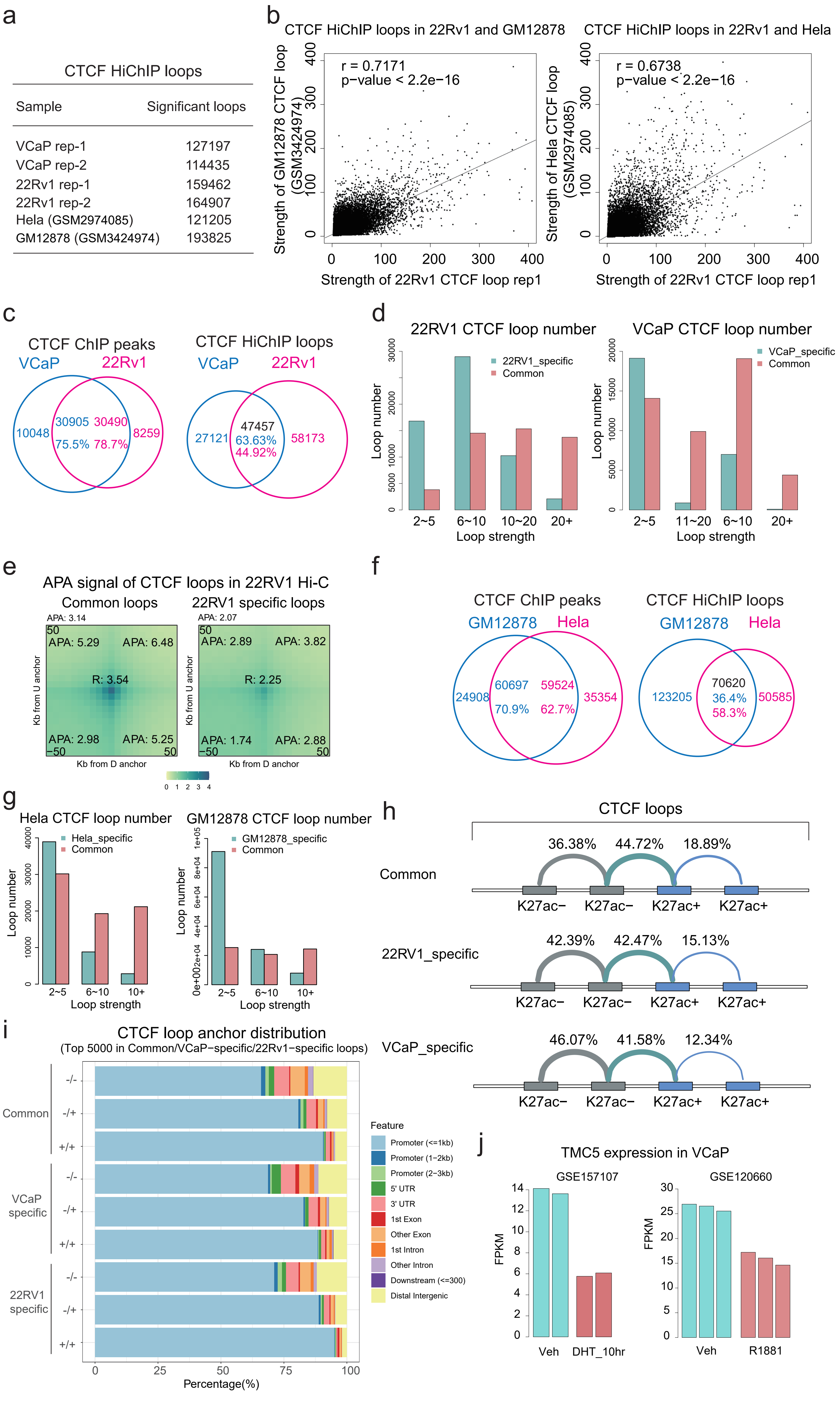

CTCF HiChIP loops

Sample	Significant loops
VCaP rep-1	127197
VCaP rep-2	114435
22Rv1 rep-1	159462
22Rv1 rep-2	164907
Hela (GSM2974085)	121205
GM12878 (GSM3424974)	193825

Supplementary Figure 3. Cell-type-specific CTCF looping analysis.

(a) The significant loop numbers of indicated CTCF HiChIP data sets. All the raw HiChIP data were processed with the same pipeline as described in the Methods section. n = 2.

(b) Scatter plots showing the association of CTCF loop strength between 22Rv1 replicate #1 and GM12878 (left); between 22Rv1 replicate #1 and Hela (right). r values and P values were calculated using Pearson correlation. n = 261712 and 209561 from left to right, respectively.

(c) Venn diagrams showing the overlap of CTCF ChIP-Seq peaks or CTCF HiChIP loops between VCaP and 22RV1 cells. The overlap ratios and peak/loop numbers were labelled with the same color as the cell names.

(d) The CTCF loop strength distribution in VCaP and 22Rv1 cells.

(e) Aggregate Peak Analysis (APA) by Juicer-tools showing the normalized 22RV1 Hi-C interaction values of the indicated CTCF HiChIP loops.

(f) Venn diagrams showing the overlap of CTCF ChIP-Seq peaks or CTCF HiChIP loops between GM12878 and Hela cells. The overlap ratio and peak/loop number were labelled by the same color of the cell name.

(g) The CTCF loop strength distribution in GM12878 and Hela cells.

(h) Summary of the classes of high-confidence interactions identified by CTCF HiChIP in VCaP and 22Rv1 cells. Light blue rectangles indicate H3K27ac+ CTCF loop anchors, which are CTCF HiChIP anchors overlapping with H3K27ac ChIP-Seq peaks. Grey blue rectangles indicate H3K27ac- CTCF loop anchors, which are CTCF HiChIP anchors without overlapping of H3K27ac ChIP-Seq peaks.

(i) Genomic distribution of indicated CTCF loop anchors. The anchor annotation was conducted by *annotatePeak* function from R package *ChIPseeker*. The promoter region was defined as 3Kb upstream and downstream of gene TSS, and further divided into <= 1Kb, 1~2Kb and 2~3Kb regions as indicated. Other genomic region information was extracted from hg19 known genes of UCSC.

(j) TMC5 expression levels in VCaP cells before and after androgen stimulation. n = 2.

Supplementary Figure 4

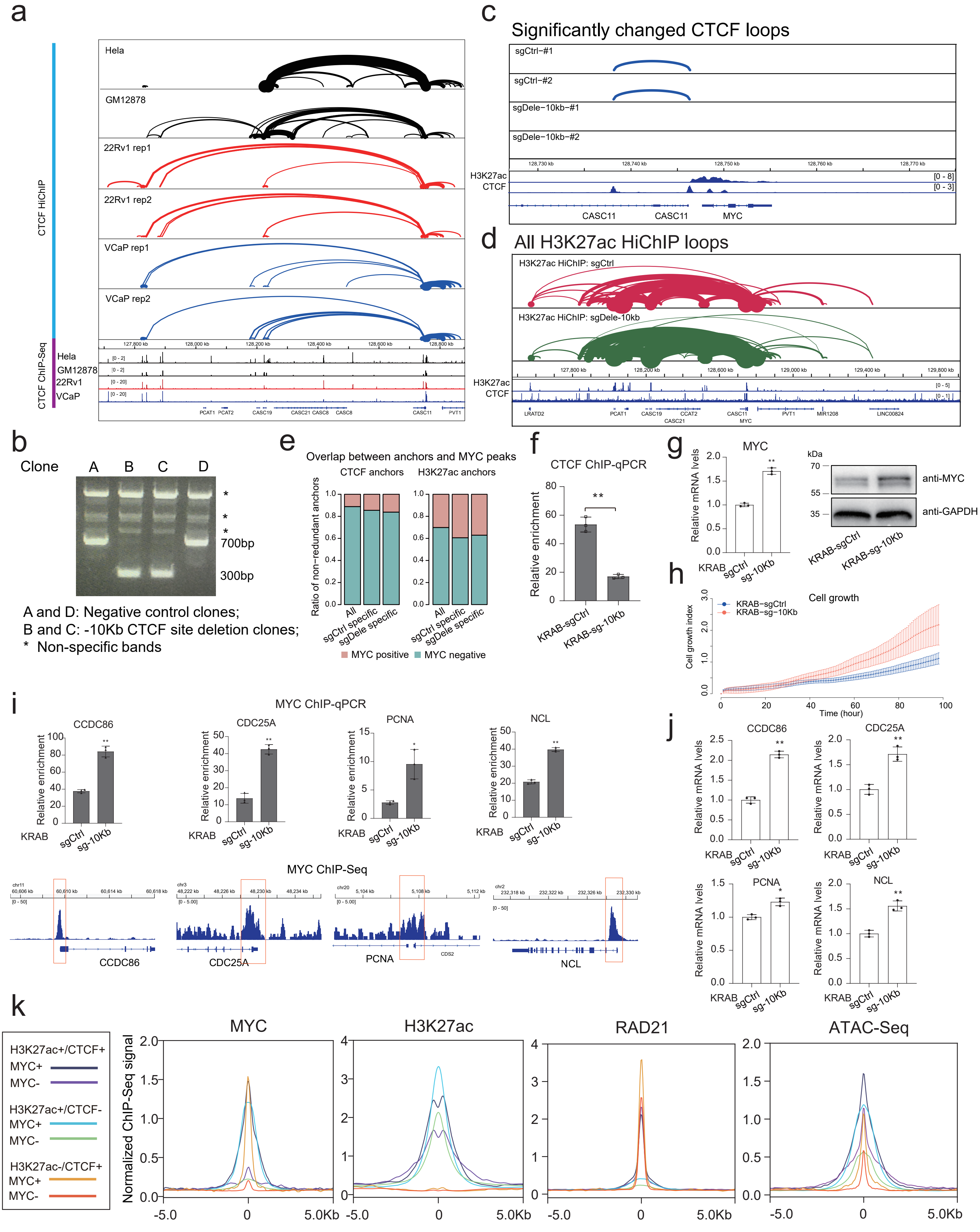

Supplementary Figure 4. MYC expression is suppressed by the -10 Kb CTCF site.

(a) CTCF loops of different cell lines in 8q24 region.

(b) The DNA gel image showing the band shift of PCR amplicons after CRISPR-mediated double knockout of the "-10 Kb CTCF site".

(c) Significantly changed CTCF loops at MYC promoter before and after "-10Kb CTCF site" deletion.

(d) All H3K27ac loops at MYC region before and after "-10Kb CTCF site" deletion.

(e) Summary of the overlap between HiChIP loop anchors and MYC binding. n = 2.

(f) CTCF ChIP-qPCR showing CTCF binding affinity changes after CRISPRi-mediated silence of the "-10 Kb CTCF site". n = 3. Data represent means ± SD. P values were calculated by two-sided Student's t test. *, P < 0.05; **, P < 0.01.

(g) MYC mRNA and protein level changes after CRISPRi-mediated silence of the "-10 Kb CTCF site". n = 3. Data represent means ± SD. P values were calculated by two-sided Student's t test. *, P < 0.05; **, P < 0.01.

(h) 22Rv1 cell proliferation with or without CRISPRi of the "-10 Kb CTCF site". The cell proliferation was monitored by a xCELLigence system (ACEA Biosciences).

(i) MYC ChIP-qPCR showing MYC binding affinity changes at four typical MYC target genes after CRISPRi of the "-10 Kb CTCF site". The red rectangles highlight the target regions for MYC ChIP-qPCR primers. n = 3. Data represent means ± SD. P values were calculated by two-sided Student's t test. *, P < 0.05; **, P < 0.01.

(j) RT-qPCR showing MYC target gene expression changes after CRISPRi-mediated silence of the "-10 Kb CTCF site". n = 3. Data represent means ± SD. P values were calculated by two-sided Student's t test. *, P < 0.05; **, P < 0.01.

(k) The aggregated MYC ChIP-Seq (22Rv1), H3K27ac ChIP-Seq (22Rv1), ATAC-Seq (22Rv1) signal and RAD21 ChIP-Seq (A549) signal at indicated anchors.

Source data are provided as a Source Data file.

Supplementary Figure 5

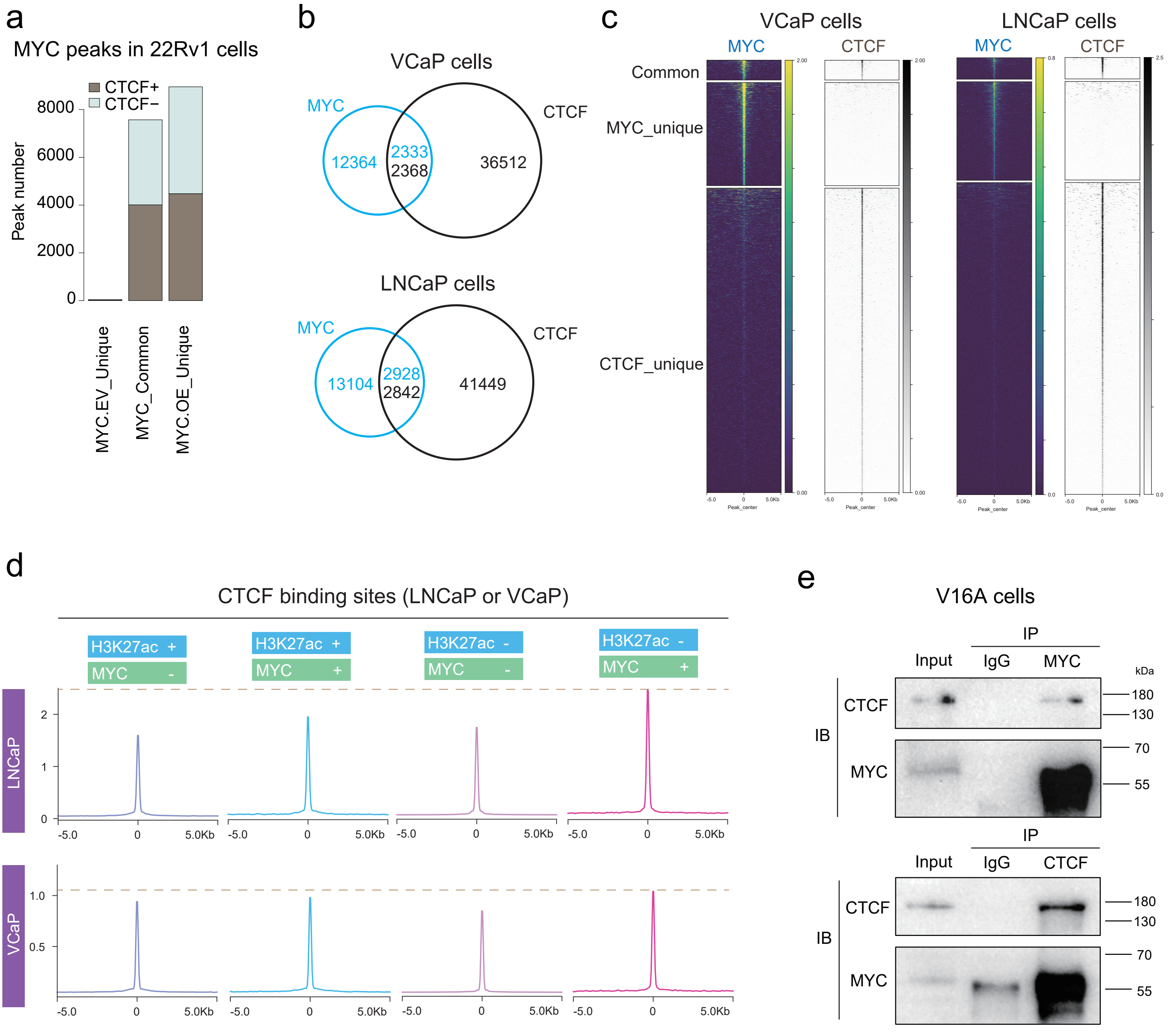

Supplementary Figure 5. Overlapping between MYC and CTCF chromatin binding sites.

(a) Number of MYC peaks with or without CTCF binding. The MYC peaks were separated into Ctrl-only, MYC-OE-only, and shared groups.

(b) Overlaps between MYC and CTCF peaks in VCaP and LNCaP cells, respectively.

(c) Heatmaps showing MYC and CTCF ChIP-Seq signal in MYC-only, CTCF-only, and shared peaks in VCaP and LNCaP cells, respectively.

(d) The aggregated CTCF ChIP-Seq signal in LNCaP and VCaP cells. CTCF peaks were divided into four groups based on MYC and H3K27ac status.

(e) Co-immunoprecipitation to detect the protein interaction between CTCF and MYC in V16A cells. This experiment was repeated independently three times (n = 3) with similar results.

Source data are provided as a Source Data file.

Supplementary Figure 6

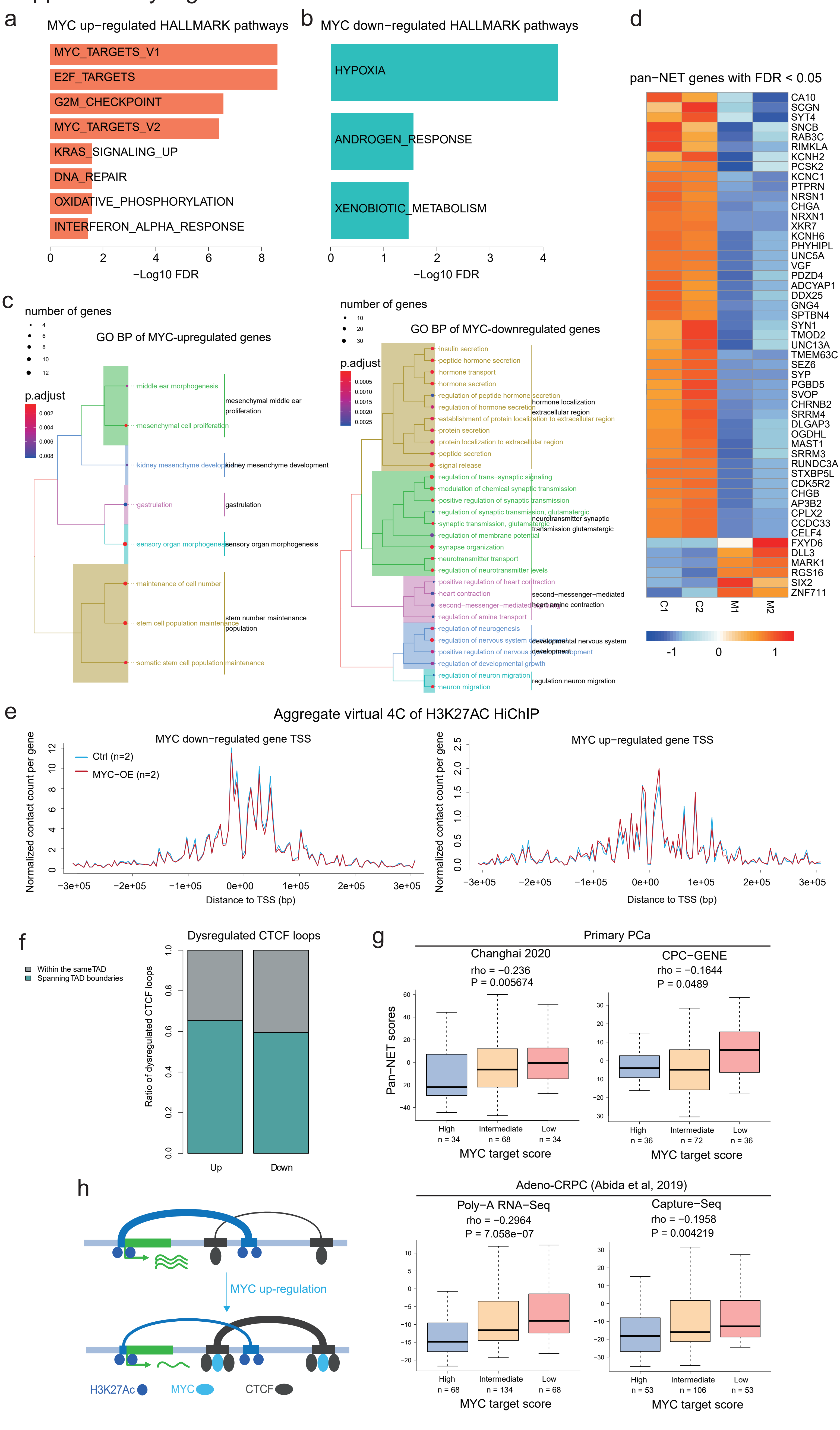

Supplementary Figure 6. MYC inhibits neuroendocrine gene expression in PCa.

(a) The upregulated MSigDB HALLMARK gene sets by GSEA of MYC-OE vs. Ctrl RNA-Seq data.

(b) The downregulated MSigDB HALLMARK gene sets by GSEA of MYC-OE vs. Ctrl RNA-Seq data.

(c) The GO BP enrichment analysis of MYC-upregulated and -downregulated genes.

(d) Heatmap showing the expression of significantly dysregulated pan-NET genes in Ctrl and MYC-OE cells.

(e) Aggregated virtual 4C analysis showing the strength of H3K27ac loops anchored at the TSSs of MYC-upregulated and -downregulated genes.

(f) Bar plot showing the ratio of MYC-regulated CTCF loops spanning TADs. n = 2.

(g) Boxplots showing the negative correlations between MYC target z-scores and pan-NET gene z-scores in primary PCa and adeno-CRPC RNA-Seq data. Box plots indicating the mean (middle line), 25th and 75th percentile (box) and 10th and 90th percentile (whiskers). From top left to top right, n = 34, 68, 34, 36, 72, 36, respectively. From bottom left to bottom right, n = 68, 134, 68, 53, 106, 53, respectively. P-values and coefficients were determined from Spearman’s rank correlation.

(h) Schematic representation of MYC-induced PCa chromatin interaction rewiring. MYC recruits more CTCF to MYC/CTCF common sites, and thus enhances the insulative CTCF-CTCF looping, resulting in the disruption of H3K27ac-associated loops and repression of target gene transcription.

Supplementary Table 1. Primers used for cloning of *MYC* gene.

Primer	DNA sequence (5'→3')[a]	Restriction enzyme
MYC-F	CCGGAATTCGCCACCATGCTGGATTTTTTTCGGGTAGTGG	*Eco*RI
MYC-R	CGCGGATCCTTACGCACAAGAGTTCCGTAGCTGT	*Bam*HI

[a] Restriction enzyme sites are underlined.

Supplementary Table 2. Primers for qPCR

qPCR-CDK5R2-F: GCCTTCCTCACCTGCCTCTAC

qPCR-CDK5R2-R: GCTCCTTGTCGGGCTCCA

qPCR-MYC-F: GGATTTTTTTCGGGTAGTGGAA

qPCR-MYC-R: ACCGAGTCGTAGTCGAGGTCAT

qPCR-GAPDH-F: ACCCACTCCTCCACCTTTGAC

qPCR-GAPDH-R: GTTGCTGTAGCCAAATTCGTTGT

qPCR-NCL-F: TCGCGAAGGCAGGTAAAAA

qPCR-NCL-R: CGACCTCTTCTCCACTGCTATCA

qPCR-PCNA-F: GTCTGAGGGCTTCGACACCTA

qPCR-PCNA-R: TGCCGGCGCATTTTAGTATT

qPCR-CCDC86-F: TCCCAGATGCTTCAGGACAAG

qPCR-CCDC86-R: GGTGACGGGCAAAGTCCTT

qPCR-CDC25A-F: AGCAACCACTGGAGGTGAAGA

qPCR-CDC25A-R: CCAATGGCCCAGGAGAATCT

Supplementary Table 3. Primers for ChIP-qPCR

ChIP-qPCR-NG-F: GCAGTTCATAAAGGCAATGTCA

ChIP-qPCR-NG-R: ACAAAAGCAGTTTGGAAAGGT

ChIP-qPCR-10kb-F: TTCGTTGCATTTGCTTTTCG

ChIP-qPCR-10kb-R: TGGGCCCCAACATCGTT

ChIP-qPCR-CDK5R2-1F: CTCCCTGAGGGCAAGGTTTAT

ChIP-qPCR-CDK5R2-1R: GGTTTTGGGCTTGTTTGGTTT

ChIP-qPCR-CDK5R2-2F: CCACTCACTTCTGTGGGCTCTA

ChIP-qPCR-CDK5R2-2R: CCCCCAGCCCAACTGAAC

ChIP-qPCR-CDK5R2-3F: CCTCCGTCCCTGTGTGCTTA

ChIP-qPCR-CDK5R2-3R: GGGACGAAGCTGAGACAACTG

ChIP-qPCR-NCL-F: TCTTCACCTCGCCACCAAGT
ChIP-qPCR-NCL-R: GGAAGTCTCGCGCGATTAGT
ChIP-qPCR-PCNA-F: CCATTCAAGGCCAACAGGAT
ChIP-qPCR-PCNA-R: GGTGGTGGCGGGAAAATC
ChIP-qPCR-CCDC86-F: AAAAAGACTGGCTCATCAATCACA
ChIP-qPCR-CCDC86-R: GCTGAGGCGGCCATGTT
ChIP-qPCR-CDC25A-F: CCTGAAGATTAAATCCAAACAAACG
ChIP-qPCR-CDC25A-R: GGTGGGAGAACAGCGAAGAC

Supplementary Table 4. sgRNA for CRISPRi

-10Kb CTCF site: TTCGGTTCCATCAATGGGTA
CDK5R2-site-1: CCAGACTGTCTGGGACGTCT
CDK5R2-site-2: AAGAGTGCCGCATCACCGAG
CDK5R2-site-3: CGCGGCCGGCGGAAAGCAAT

Supplementary Table 5. Primers for 3C

qA: GCAATGGGTCATGCCTGTAA
qC: TGGCCAGAACTTGCAATACTATGT
qD: CAGCACAGCCACTCTCTTCCT
qB-anchor: TCATAGGCTGTGGGCACC
qNC1: CATGCATCTGTGTTCCCAGCTA
qNC2: CCCCAAACAGCCACTCTTAACT
Internal control-F: CGATCCATCATCCGCAATG
Internal control-R: AGCCAAGCTCAGCGCAAC
PST-I-A-F: GAGAGGAAAGAAGACACAGAGGAAATG
PST-I-A-R: CTGTGGGTAAAGACAATAGGCAAGTG
PST-I-B-F: ACTGCAAAGGAAACAACGGCGTA
PST-I-B-R: ACGGGCAGGTAGGTGGGTC
PST-I-C-F: CAAGGCTGGTTCAACATACACAAATC
PST-I-C-R: TAAGGAGAGTTTGGGCTGAGATGATG
PST-I-D-F: ACTAAGCAAGGGAGTAAGGCTGGAG
PST-I-D-R: TTACTTTCACAGCACAGCCACTCTC
PST-I-NC1-F: GGTCTGACTCTATCACCCAGGCTG
PST-I-NC1-R: ACACTTTGGGTGGTTGAGGTAGGAG
PST-I-NC2-F: GACCATCCTGGCTAACAAGGTGAAAC
PST-I-NC2-R: CTGCTGGTTTACACTGTTTCTTCAAG

Supplementary Fig. 4b

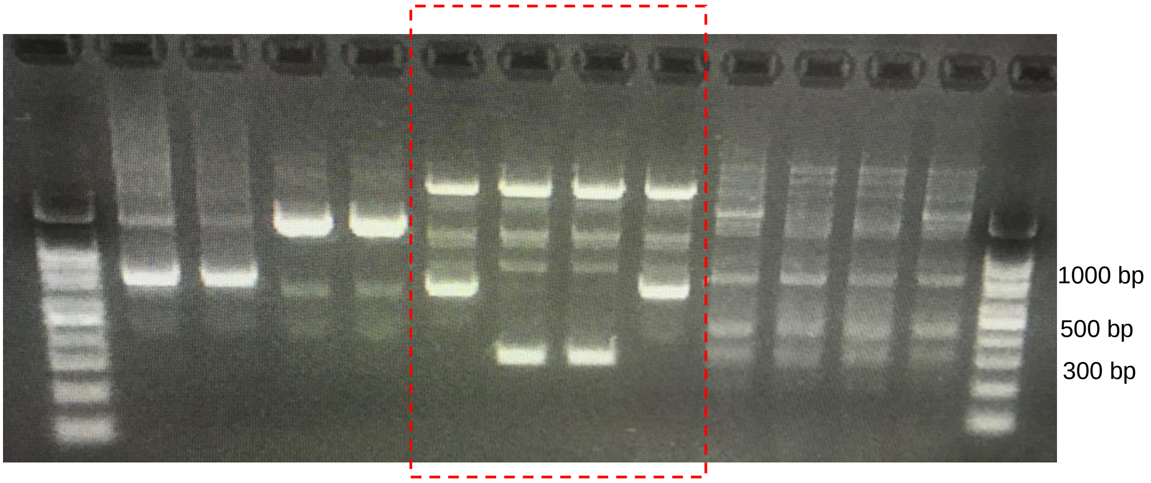

Supplementary Fig. 4g

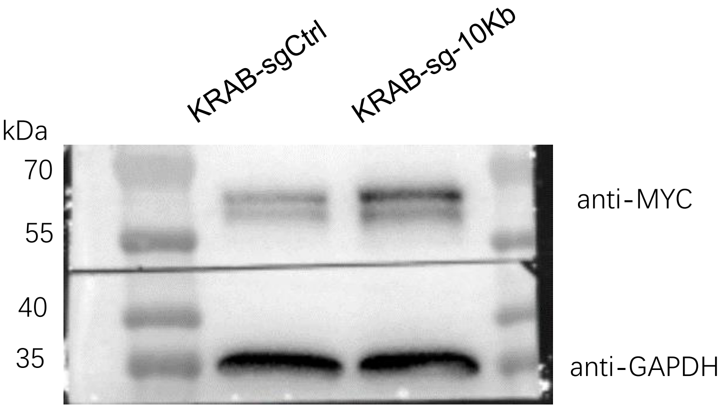

Supplementary Fig. 5e

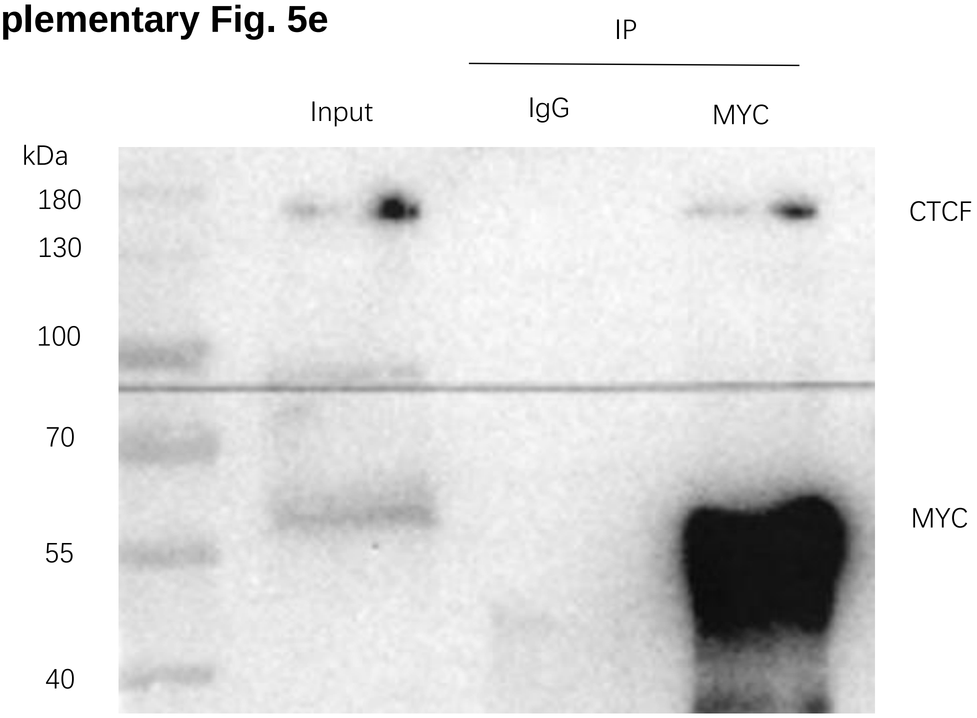

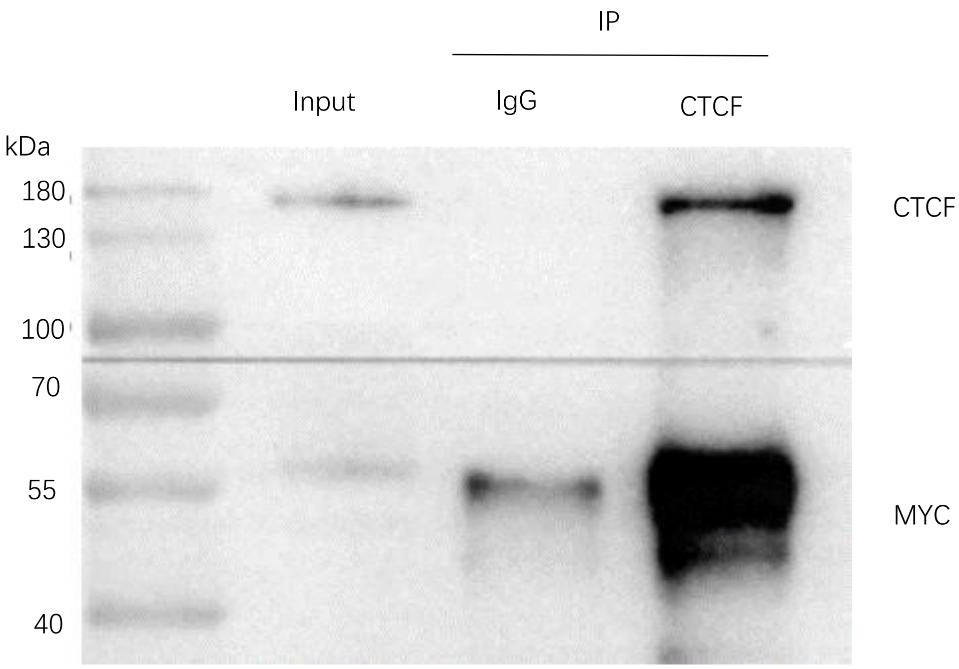

Supplement: Supplementary file 1 — Supplementary Information [file 41467_2023_37544_MOESM1_ESM.pdf]
