# Supplementary material for: MYC reshapes CTCF-mediated chromatin architecture in prostate cancer
Source: Nat Commun. 2023 Mar 30;14:1787. doi: 10.1038/s41467-023-37544-3 (PMC10063626; doi:10.1038/s41467-023-37544-3)
Supplement: Supplementary file 3 — Description of Additional Supplementary Files [file 41467_2023_37544_MOESM3_ESM.pdf]

Description of Additional Supplementary Files

File Name: Supplementary Data 1
Description: Top 20 motifs enriched in PRAD ATAC-Seq peaks located in distal enhancers looped to promoters of genes (with both AR and H3K27ac loops) up-regulated by DHT 2hr.

File Name: Supplementary Data 2
Description: Top 20 motifs enriched in PRAD ATAC-Seq peaks located in distal enhancers looped to promoters of genes (with H3K27ac but not AR loops) down-regulated by DHT 2hr.
