# Supplementary material for: MYC reshapes CTCF-mediated chromatin architecture in prostate cancer
Source: Nat Commun. 2023 Mar 30;14:1787. doi: 10.1038/s41467-023-37544-3 (PMC10063626; doi:10.1038/s41467-023-37544-3)
Supplement: Supplementary file 6 — Reporting Summary [file 41467_2023_37544_MOESM6_ESM.pdf]

nature portfolio

Corresponding author(s): Haiyang Guo
Last updated by author(s): Mar 17, 2023

Reporting Summary

Nature Portfolio wishes to improve the reproducibility of the work that we publish. This form provides structure for consistency and transparency in reporting. For further information on Nature Portfolio policies, see our Editorial Policies and the Editorial Policy Checklist.

Statistics

For all statistical analyses, confirm that the following items are present in the figure legend, table legend, main text, or Methods section.

n/a	Confirmed	
☐	☒	The exact sample size (*n*) for each experimental group/condition, given as a discrete number and unit of measurement
☐	☒	A statement on whether measurements were taken from distinct samples or whether the same sample was measured repeatedly
☐	☒	The statistical test(s) used AND whether they are one- or two-sided<br>*Only common tests should be described solely by name; describe more complex techniques in the Methods section.*
☐	☒	A description of all covariates tested
☐	☒	A description of any assumptions or corrections, such as tests of normality and adjustment for multiple comparisons
☐	☒	A full description of the statistical parameters including central tendency (e.g. means) or other basic estimates (e.g. regression coefficient) AND variation (e.g. standard deviation) or associated estimates of uncertainty (e.g. confidence intervals)
☐	☒	For null hypothesis testing, the test statistic (e.g. *F*, *t*, *r*) with confidence intervals, effect sizes, degrees of freedom and *P* value noted<br>*Give P values as exact values whenever suitable.*
☒	☐	For Bayesian analysis, information on the choice of priors and Markov chain Monte Carlo settings
☒	☐	For hierarchical and complex designs, identification of the appropriate level for tests and full reporting of outcomes
☐	☒	Estimates of effect sizes (e.g. Cohen's *d*, Pearson's *r*), indicating how they were calculated

Our web collection on statistics for biologists contains articles on many of the points above.

Software and code

Policy information about availability of computer code

Data collection	No software was used to collect data in this study.
Data analysis	R (version 4.2.2), STAR (version 2.4.2a), BOWTIE2 (version 2.2.1), MACS2 (v2.2.7.1), samtools (v.0.1.18), Deeptools (v3.4.3), UCSC tools (v385), IGV (v2.8.12), Juicer (v1.6), hichipper(v0.7.7) and HiCUP (v0.7.2) were used for data analysis in this study.

For manuscripts utilizing custom algorithms or software that are central to the research but not yet described in published literature, software must be made available to editors and reviewers. We strongly encourage code deposition in a community repository (e.g. GitHub). See the Nature Portfolio guidelines for submitting code & software for further information.

Data

Policy information about availability of data

All manuscripts must include a data availability statement. This statement should provide the following information, where applicable:

- Accession codes, unique identifiers, or web links for publicly available datasets
- A description of any restrictions on data availability
- For clinical datasets or third party data, please ensure that the statement adheres to our policy

Hg19 human reference genome was downloaded from NCBI [https://www.ncbi.nlm.nih.gov/assembly/GCF_000001405.13/].The following publicly available ChIP-Seq and ATAC-Seq data sets used in this paper were obtain from GEO: LNCaP H3K27ac ChIP-Seq (GSM1249448)53 [https://www.ncbi.nlm.nih.gov/geo/query/acc.cgi?acc=GSM1249448], 22Rv1 H3K27ac ChIP-Seq (GSM2827407)18 [https://www.ncbi.nlm.nih.gov/geo/query/acc.cgi?acc=GSM2827407], 22Rv1 CTCF ChIP-Seq

(GSM2828839)18 [https://www.ncbi.nlm.nih.gov/geo/query/acc.cgi?acc=GSM2828839], VCaP AR ChIP-Seq (GSE55062)54 [https://www.ncbi.nlm.nih.gov/geo/query/acc.cgi?acc=GSE55062], VCaP CTCF ChIP-Seq (GSE84432) 55 [https://www.ncbi.nlm.nih.gov/geo/query/acc.cgi?acc=GSE84432], GM12878 CTCF ChIP-Seq (GSM935611)18 [https://www.ncbi.nlm.nih.gov/geo/query/acc.cgi?acc=GSM935611], Hela CTCF ChIP-Seq (GSM2915166)16 [https://www.ncbi.nlm.nih.gov/geo/query/acc.cgi?acc=GSM2915166], A549 RAD21 ChIP-Seq (GSM3106369)18 [https://www.ncbi.nlm.nih.gov/geo/query/acc.cgi?acc=GSM3106369] and 22Rv1 ATAC-Seq (GSM3075372)56 [https://www.ncbi.nlm.nih.gov/geo/query/acc.cgi?acc=GSM3075372]. To determine the relative peak binding affinities among cell lines, the CTCF ChIP-Seq data of 22Rv1 [https://www.encodeproject.org/experiments/ENCSR857PBV/], C4-2B [https://www.encodeproject.org/experiments/ENCSR460LGH/], LNCaP [https://www.encodeproject.org/experiments/ENCSR315NAC/] and VCaP [https://www.encodeproject.org/experiments/ENCSR265ARE/] cells and H3K27ac ChIP-Seq data of 22Rv1 and VCaP cells were also downloaded from ENCODE (https://www.encodeproject.org). The RNA-Seq data of 22Rv1, C4-2B, LNCaP and VCaP cells were obtained from CCLE (https://portals.broadinstitute.org/ccle/home) and GEO (GSE25183)57 [https://www.ncbi.nlm.nih.gov/geo/query/acc.cgi?acc=GSE25183]. The combined ATAC-Seq peaks (hg38) of TCGA-PRAD samples were downloaded from GDC44 and lifted over to hg19 by UCSC 'hg38ToHg19.over.chain'. RNA-Seq and DNA methylation data of Changhai 2020 PCa cohort58 were obtained from www.cpgea.com. GM12878 CTCF and Hela CTCF HiChIP data were obtained from GEO by accession numbers GSM342497417 [https://www.ncbi.nlm.nih.gov/geo/query/acc.cgi?acc=GSM3424974] and GSM297408516 [https://www.ncbi.nlm.nih.gov/geo/query/acc.cgi?acc=GSM2974085], respectively. The RNA-Seq, H3K27ac ChIP-Seq and MYC ChIP-Seq data of VCaP cells we generated in another study were deposited to GEO under GSE15710414 [https://www.ncbi.nlm.nih.gov/geo/query/acc.cgi?acc=GSE157104]. All the RNA-Seq, ChIP-Seq and HiChIP data we generated for this study were deposited to GEO under GSE172498 [https://www.ncbi.nlm.nih.gov/geo/query/acc.cgi?acc=GSE172498] and GSE200168 [https://www.ncbi.nlm.nih.gov/geo/query/acc.cgi?acc=GSE200168]. Source data are provided with this paper. The remaining data are available within the Article, Supplementary Information or Source Data file.

Human research participants

Policy information about studies involving human research participants and Sex and Gender in Research.

Reporting on sex and gender	N/A
Population characteristics	N/A
Recruitment	N/A
Ethics oversight	N/A

Note that full information on the approval of the study protocol must also be provided in the manuscript.

Field-specific reporting

Please select the one below that is the best fit for your research. If you are not sure, read the appropriate sections before making your selection.

☒ Life sciences ☐ Behavioural & social sciences ☐ Ecological, evolutionary & environmental sciences

For a reference copy of the document with all sections, see nature.com/documents/nr-reporting-summary-flat.pdf

Life sciences study design

All studies must disclose on these points even when the disclosure is negative.

Sample size	Two replicates for RNA-Seq, ChIP-Seq and HiChIP experiments in 22Rv1 cells with or without MYC overexpression. The sample sizes were chosen based on the general experiences in this field.
Data exclusions	No data was excluded from the analyses.
Replication	Experiment were repeated independently three times with similar results.
Randomization	Randomization was not relevant to our study as our experiments only include technique replicates.
Blinding	The investigators were blinded during data collection but not blinded during data analysis as our experiments only include technique replicates.

Reporting for specific materials, systems and methods

We require information from authors about some types of materials, experimental systems and methods used in many studies. Here, indicate whether each material, system or method listed is relevant to your study. If you are not sure if a list item applies to your research, read the appropriate section before selecting a response.

Materials & experimental systems

n/a	Involved in the study
☐	☒ Antibodies
☐	☒ Eukaryotic cell lines
☒	☐ Palaeontology and archaeology
☒	☐ Animals and other organisms
☒	☐ Clinical data
☒	☐ Dual use research of concern

Methods

n/a	Involved in the study
☐	☒ ChIP-seq
☒	☐ Flow cytometry
☒	☐ MRI-based neuroimaging

Antibodies

Antibodies used

MYC Abcam ab32072, Rabbit monoclonal [Y69], Lot: GR3377350-5 (ChIP, 5 μg; IP, 2 ug; Western blotting, 1:1,000)
MYC Santa Cruz sc-40, Mouse monoclonal [9E10], Lot: K1920 (PLA, 1:50)
CTCF Cell Signaling Technology 3418S, Rabbit monoclonal [D31H2], Lot: 5 (Hi-ChIP, 6 μg; ChIP, 5 ug; IP, 2 μg; PLA, 1:400; Western blotting, 1:1,000)
H3K27ac Abcam ab4729, Rabbit polyclonal, Lot: GR3374555-1 (ChIP, 5 μg; Western blotting, 1:1,000)
AR Abcam ab108341, Rabbit monoclonal [ER179(2)], Lot: GR3233427-1 (ChIP, 5 μg)
GAPDH Cell Signaling Technology 5174S, Rabbit monoclonal [D16H11], Lot: 8 (Western blotting, 1:1,000)
GFP Abcam ab290, Rabbit polyclonal, Lot: GR3321575-1 (IP, 2 μg; Western blotting, 1:2,000)
Flag Cell Signaling Technology 14793S, Rabbit monoclonal [D6W5B], Lot: 7 (Western blotting, 1:1,000)
MAX Abcam ab199489, Rabbit monoclonal [EPR19352], Lot: GR3441065-2 (Western blotting, 1:1,000)
Rabbit mAb IgG Cell Signaling Technology 3900S, Rabbit monoclonal [DA1E], Lot: 45 (IP, 2 μg)
Mouse anti-rabbit IgG mAb (HRP Conjugate) Cell Signaling Technology 93702S, Mouse monoclonal [D4W3E], Lot: 5 (Western blotting, 1:1,000)

Validation

All antibodies used in this study are commercially validated and all are validated by the vendors for the specific assays and species used. A full reference list can be found on the official website of the manufacturer.
1. MYC Abcam ab32072: reacts with mouse, rat, human; applications for Flow Cyt (Intra), WB, ICC/IF, ChIC/CUT&RUN-seq, ChIP-sequencing, IHC-P, IP were validated on the manufacturer's website.
https://www.abcam.com/products/primary-antibodies/c-myc-antibody-y69-chip-grade-ab32072.html
2. MYC Santa Cruz sc-40: reacts with mouse, rat, human, monkey, feline and canine; applications for WB, IP, IF, IHC(P), FCM, ELISA were validated on the manufacturer's website.
https://www.scbt.com/p/c-myc-antibody-9e10
3. CTCF Cell Signaling Technology 3418S: reacts with mouse, rat, human and monkey; applications for WB, IP, IF, IHC, ChIP, CUT&RUN were validated on the manufacturer's website. https://www.cellsignal.com/products/primary-antibodies/ctcf-d31h2-xp-rabbit-mab/3418
4. H3K27ac Abcam ab4729: reacts with mouse, rat, cow, human, recombinant fragment; applications for ICC/IF, WB, IHC-P, ChIP, PepArr were validated on the manufacturer's website.
https://www.abcam.com/products/primary-antibodies/histone-h3-acetyl-k27-antibody-chip-grade-ab4729.html
5. AR Abcam ab108341: reacts with mouse, rat, human; applications for ICC/IF, WB, IHC-P, ChIP were validated on the manufacturer's website.
https://www.abcam.com/products/primary-antibodies/androgen-receptor-antibody-er1792-chip-grade-ab108341.html
6. GAPDH Cell Signaling Technology 5174S: reacts with mouse, rat, human and monkey; applications for IF, WB, IHC were validated on the manufacturer's website.
https://www.cellsignal.com/products/primary-antibodies/gapdh-d16h11-xp-rabbit-mab/5174
7. GFP Abcam ab290: reacts with species independent; applications for ELISA, IHC-Fr, ICC, IHC-P, IP, WB, IHC-FoFr, IHC-FrFl, Electron Microscopy were validated on the manufacturer's website.
https://www.abcam.com/products/primary-antibodies/gfp-antibody-ab290.html
8. MAX Abcam ab199489: reacts with mouse, rat, humant; applications for ChIP-seq, IP, WB were validated on the manufacturer's website.
https://www.abcam.com/products/primary-antibodies/max-antibody-epr19352-chip-grade-ab199489.html
9. Rabbit mAb IgG Cell Signaling Technology 3900S: Rabbit (DA1E) mAb IgG XP Isotype Control is not directed against any known antigen; it functions as an isotype control for rabbit IgG antibodies; applications for ChIP, IP, IHC, IF, Flow Cytometry were validated on the manufacturer's website.
https://www.cellsignal.com/products/primary-antibodies/rabbit-da1e-mab-igg-xp-isotype-control/3900

Eukaryotic cell lines

Policy information about cell lines and Sex and Gender in Research

Cell line source(s)

VCaP ATCC CRL-2876
22Rv1 ATCC CRL-2505
HEK293FT Thermo Fisher Scientific, R70007
V16A was established by Dr. Amina Zoubeidi's laboratory.

Authentication

We utilized ATCC services following extended passages to authenticate by utilizing Short Tanden Repeat (STR) profiling.

Mycoplasma contamination

We utilized ATCC services following extended passages to authenticate by utilizing Short Tanden Repeat (STR) profiling.

March 2021

Mycoplasma contamination

Sequences were amplified 17 STR loci plus Amelogenin using Promega's PowerPlex® 18D System. A comprehensive analysis report interprets both karyotypically normal and abnormal cell lines, includes a electropherograms supporting the allele calls at each locus, known reference profiling against the ATCC STR database and a comprehensive interpretation of results. All cell lines used in this study were tested negative for mycoplasma contamination

Commonly misidentified lines
(See ICLAC register)

No cell lines used in this study are listed in the database of commonly misidentified cell lines maintained by ICLAC.

ChIP-seq

Data deposition

☒ Confirm that both raw and final processed data have been deposited in a public database such as GEO.

☒ Confirm that you have deposited or provided access to graph files (e.g. BED files) for the called peaks.

Data access links
May remain private before publication.

https://www.ncbi.nlm.nih.gov/geo/query/acc.cgi?acc=GSE200168
All ChIP-Seq data we generated for this study were deposited to GEO under GSE200168, and the access tokens for reviewers is mnchgcuslnmzxut

Files in database submission

GSM6016304 ChIP-CTCF-Con1
GSM6016305 ChIP-CTCF-Con2
GSM6016306 ChIP-CTCF-MYC1
GSM6016307 ChIP-CTCF-MYC2
GSM6016308 ChIP-H3K27ac-Con1
GSM6016309 ChIP-H3K27ac-Con2
GSM6016310 ChIP-H3K27ac-MYC1
GSM6016311 ChIP-H3K27ac-MYC2
GSM6016312 ChIP-Input-Con1
GSM6016313 ChIP-Input-Con2
GSM6016314 ChIP-Input-MYC1
GSM6016315 ChIP-Input-MYC2
GSM6016316 ChIP-MYC-Con1
GSM6016317 ChIP-MYC-Con2
GSM6016318 ChIP-MYC-MYC1
GSM6016319 ChIP-MYC-MYC2

Genome browser session
(e.g. UCSC)

https://genome-asia.ucsc.edu/s/Haiyang/hg19_CTCF_ChIP%2DSeq

Methodology

Replicates

Two replicates for each group

Sequencing depth

Sample Total_reads Mapped_reads Read_type Read_length
CTCF_Con1 129530692 124194660 PE 150 bp
CTCF_Con2 85420034 82568650 PE 150 bp
CTCF_MYC1 100319958 95924250 PE 150 bp
CTCF_MYC2 82517676 78343559 PE 150 bp
H3K27ac_Con1 99883928 98060560 PE 150 bp
H3K27ac_Con2 88441794 86856202 PE 150 bp
H3K27ac_MYC1 79411620 77992277 PE 150 bp
H3K27ac_MYC2 99488564 97632115 PE 150 bp
MYC_Con1 100326086 88547456 PE 150 bp
MYC_Con2 96983970 84676837 PE 150 bp
MYC_MYC1 93860660 77101135 PE 150 bp
MYC_MYC2 87235142 76246345 PE 150 bp
Input_Con1 124260354 112344510 PE 150 bp
Input_Con2 119307534 107059716 PE 150 bp
Input_MYC1 120478470 102503668 PE 150 bp
Input_MYC2 107200404 97133353 PE 150 bp

Antibodies

anti-MYC (ab32072, Abcam)
anti-CTCF (3418S, CST)
anti-H3K27ac (ab4729, Abcam)

Peak calling parameters

```
bowtie2 -p 30 -x /home/Homo_sapiens_hg19/Ensembl/GRCh37/Sequence/Bowtie2Index/genome -1 ${id}_R1.fq.gz -2 ${id}_R2.fq.gz 2> ./bowtie2_result/$id.log | samtools sort -O bam -@ 30 -o ./bowtie2_result/$id.sort.bam

macs2 callpeak  -t $a -c ${input} --keep-dup=1 -g hs -B --SPMR --name=${a}_SPMR

macs2 bdgcmp -t  ${a}_SPMR_treat_pileup.bdg -c ${a}_SPMR_control_lambda.bdg  -o ${a}_SPMR_FE.bdg -m FE
```

Data quality

Number of peaks with q-value < 0.05 after removing ENCODE blacklist regions:

Data quality

1 CTCF_Con1 76087
2 CTCF_Con2 74924
3 CTCF_MYC1 73694
4 CTCF_MYC2 73508
5 H3K27ac_Con1 67275
6 H3K27ac_Con2 64714
7 H3K27ac_MYC1 62167
8 H3K27ac_MYC2 63063
9 MYC_Con1 11808
10 MYC_Con2 8999
11 MYC_MYC1 20965
12 MYC_MYC2 19844

Software

BOWTIE2, samtools and MACS2 were used for ChIP-Seq data analysis. The ChIP-Seq data reported in this paper have been deposited to GEO under accession number GSE200168.
